# An Immunometabolic Shift Modulates Cytotoxic Lymphocyte Activation During Melanoma Progression in TRPA1 Channel Null Mice

**DOI:** 10.3389/fonc.2021.667715

**Published:** 2021-05-10

**Authors:** Maria Fernanda Forni, Omar Alberto Domínguez-Amorocho, Leonardo Vinícius Monteiro de Assis, Gabriela Sarti Kinker, Maria Nathalia Moraes, Ana Maria de Lauro Castrucci, Niels Olsen Saraiva Câmara

**Affiliations:** ^1^ Laboratory of Transplantation Immunobiology, Institute of Biomedical Sciences, University of São Paulo, São Paulo, Brazil; ^2^ Laboratory of Comparative Physiology of Pigmentation, Department of Physiology, Institute of Biosciences, University of São Paulo, São Paulo, Brazil; ^3^ Laboratory of Translational Immuno-Oncology A. C. Camargo Cancer Center – International Research Center, São Paulo, Brazil; ^4^ Laboratory of Neurobiology, Department of Physiology and Biophysics, Institute of Biomedical Sciences, University of São Paulo, São Paulo, Brazil; ^5^ Department of Biology, University of Virginia, Charlottesville, VA, United States

**Keywords:** TRPA1 channel, melanoma, immunometabolism, CD8+ T cells, metabolic shift

## Abstract

Melanoma skin cancer is extremely aggressive with increasing incidence and mortality. Among the emerging therapeutic targets in the treatment of cancer, the family of transient receptor potential channels (TRPs) has been reported as a possible pharmacological target. Specifically, the ankyrin subfamily, representing TRPA1 channels, can act as a pro-inflammatory hub. These channels have already been implicated in the control of intracellular metabolism in several cell models, but little is known about their role in immune cells, and how it could affect tumor progression in a process known as immune surveillance. Here, we investigated the participation of the TRPA1 channel in the immune response against melanoma tumor progression in a mouse model. Using *Trpa1* ^+/+^ and *Trpa1* ^-/-^ animals, we evaluated tumor progression using murine B16-F10 cells and assessed isolated CD8+ T cells for respiratory and cytotoxic functions. Tumor growth was significantly reduced in *Trpa1* ^-/-^ animals. We observed an increase in the frequency of circulating lymphocytes. Using a dataset of CD8+ T cells isolated from metastatic melanoma patients, we found that *TRPA1* reduction correlates with several immunological pathways. Naïve CD8+ T cells from *Trpa1* ^+/+^ and *Trpa1* ^-/-^ animals showed different mitochondrial respiration and glycolysis profiles. However, under CD3/CD28 costimulatory conditions, the absence of TRPA1 led to an even more extensive metabolic shift, probably linked to a greater *in vitro* killling ability of *Trpa1* ^-/-^ CD8+ T cells. Therefore, these data demonstrate an unprecedented role of TRPA1 channel in the metabolism control of the immune system cells during carcinogenesis.

## Introduction

Melanocytes are key players in skin biology since they produce a pigment, melanin, that protects the skin against the deleterious effects of UV radiation and visible light (1, 2). However, the uncontrolled and deregulated proliferation of melanocytes may result in cutaneous, mucosal, or uveal melanoma. Cutaneous melanoma (CM) is the most aggressive and treatment-resistant skin cancer (3, 4), being responsible for the majority of deaths, thus imposing a massive economic burden on the health system (3–5).

The interaction of cancer cells with cells and molecules or metabolites in tissues, also known as tumor microenvironment, plays an important role in cancer progression (6). Among these components, immune cells are responsible for the immune surveillance (7–9). They are directly involved in the tumor microenvironment and may favor or halt cancer development (10). During early stages of cancer, effector immune cells efficiently eliminate immunogenic cancer cells; however, selected cancer cells that survive, can progress, and evolve to clinically detectable tumors, through several cellular mechanisms that lead to evasion or inactivation of immune cells (10).

Due to this fact, new pharmacological targets for melanoma treatment are greatly needed. Among these new players, transient receptor potential (TRP) channels have received some attention as putative targets for pain and diabetes treatment, skin, central nervous and cardiovascular disorders [reviewed in (11–15)]. TRP channels are divided into six subfamilies, among them, TRPA (“A” for ankyrin) (16). Several *in vitro* and *in vivo* studies have shown the involvement of TRPC, TRPV, and TRPM family in many cancer models. However, the clinical and therapeutic value of TRP channels is still elusive (17).

Recently, it became evident that activation, growth and proliferation, engagement of effector functions, and homeostasis of immune cells are intimately linked and dependent on dynamic changes in cellular metabolism (18). This is even more evident in the tumor microenvironment where the competition for nutrients conditioning metabolic reprogramming can impact T cell activation and function (19–21). Moreover, even though TRPA1 has been associated with the control of growth, survival, and activation of neutrophils, macrophages, B and T cells, the roles of this channel upon the metabolic regulation of T lymphocyte activation remain unclear, especially in the tumor microenvironment (22, 23).

In an attempt to fill this gap, in this study we sought to evaluate the contribution of TRPA1 for the metabolic activation of the immune system and its impact on the carcinogenic process in a murine model of melanoma cancer. Through the usage of intact cell metabolic evaluation and flow cytometry, we demonstrated that the lack of TRPA1 in CD8+ T cells leads to increased respiratory response and glycolysis that culminates with T cell activation and enhanced killing activity. This study provides a novel evidence that TRPA1 could represent an important modulator of immune cells and a putative new pharmacological target in melanoma treatment.

## Material and Methods

### *In Vivo* Procedures

All experimental procedures were performed according to Brazilian legislation approved by the Committee for Animal Use (CEUA IB/USP, number 255/2016, 14^th^ of June 2016). Experiments were performed on B6;129 (*Trpa1^+/+^*) male mice, which is the result of 129 SvWT crossing with C57BL/6J, both provided by the Institute of Biomedical Sciences vivarium, University of São Paulo, originally acquired from Jackson Laboratories and on *Trpa1^-/-^* male mice in a mix background (B6;129), also acquired from Jackson Laboratories (B6;129P-Trpa1^tm1Kykw^/J, 003770). Three to eight month-old *Trpa1^+/+^* and *Trpa1^-/-^* animals were used. Mouse genotypes were confirmed according to the instructions provided by Jackson Laboratories.

Mice were kept under a 12:12 light/dark cycle (800 – 1000 lux white LED light, ranging from 420 to 750 nm) at controlled temperature (22 ± 2°C). Lights were on at 7 a.m. and off at 7 p.m. Mice were subcutaneously inoculated in the right flank with 2x10^6^ B16-F10 cells (kindly donated by Prof. Roger Chammas, Faculty of Medicine, University of São Paulo) in 100 µL of phosphate buffered saline (PBS). Control animals were injected with the same volume of PBS. Then, mice were single housed for the entire experiment. Animals were euthanized with CO_2_, 22 days after inoculation, and death was assured by cervical dislocation. After euthanasia every animal was visually inspected and no metastasis was found, as previously demonstrated (24). The organs and blood were harvested and immediately processed or stored at -80^o^C as described below.

### Mouse Weight, Food Consumption, and Tumor Volume

All the following parameters were assessed every 3 to 4 days at the same time of the day (from 2 to 3 p.m.). Mouse weight values were expressed in grams. On the 22^nd^ day, tumor was resected and weighed. Food consumption was assessed by measuring the initial and every 3 to 4 days the weight of ration pellets and expressed in grams. Tumor volume (mm^3^) was evaluated from the 13^th^ day onwards, measuring length, width, and height with a caliper rule, and calculated following the formula: π/6 x length x width x height (25). Before the 13th day tumor growth was considered negligible due to the absence of visible growth, and thus, plotted as zero.

### Gene Expression

Small fragments of tumor were homogenized in TRIzol (Thermo Fisher Scientific, USA) and total RNA was extracted and purified according to the kit manufacturer’s instructions (Direct-zol™ RNA MiniPrep, Zymo Research, USA). RNA concentration (OD_260_) was determined in a spectrophotometer (Nanodrop, USA), and 1 µg was subject to reverse transcription with SuperScript III Reverse Transcriptase, random hexamer primers and other reagents, according to the manufacturer’s instructions (Thermo Fisher Scientific, USA), as described previously (26). To evaluate gene expression, 25 ng of cDNA per well were subject to quantitative PCR (qPCR) using species-specific primers (**Table 1**) spanning introns, based on sequences obtained from GenBank (http://www.ncbi.nlm.nih.gov/genbank), designed by Primer Blast (http://www.ncbi.nlm.nih.gov/genbank) and synthesized by IDT (Integrated DNA Technologies, USA) or Exxtend (Brazil). *Rpl37a* RNA was used to normalize gene expression values, which has been previously shown to be an adequate housekeeping gene in melanoma tumor samples (24). Reactions were carried out using BioRad iQ™ SYBR^®^ Green Supermix (Bio-Rad Laboratories, USA) with the following conditions in iQ5 thermocycler (Bio-Rad Laboratories, USA): 3 min at 95°C, followed by 45 cycles of 15 s at 95°C, 30 s at 60°C, and 80 cycles of 10 s at 55°C with a gradual rise of 0.5°C. Negative controls with no templates were routinely included. Gene expression was quantified according to the 2^ΔΔCt^ method (27). ΔC_t_ was determined by subtracting the normalizer C_t_ from the C_t_ of the gene of interest at the same time point, both corresponding to the average of duplicates of the same cDNA sample. The mean value obtained from control mice was subtracted from all other values, obtaining the ΔΔC_t_, which was used as a negative exponential of base 2 (2^-ΔΔCt^). The log values were obtained from a minimal of three animals of at least two independent experiments. Data are shown as the mean ± SD.

**Table 1:** List of primers used (300 nM) in the manuscript, and the corresponding access numbers.

Gene	Forward Sequence 5’ – 3’	Reverse Sequence 5’ – 3’
*Rpl37a* NM_009084.4	GCATGAAAACAGTGGCCGGT	AGGGTCACACAGTATGTCTCAAAA
*Il-1-β* NM_008361	GCAACTGTTCCTGAACTCAACT	ATCTTTTGGGGTCCGTCAACT
*Il-6* NM_010559	CCTGAGACTCAAGCAGAAATGG	AGAAGGAAGGTCGGCTTCAGT
*Il-10* NM_010548	GCTCTTACTGACTGGCATGAG	CGCAGCTCTAGGAGCATGTG
*Il-12* NM_008351.2	CTGTGCCTTGGTAGCATCTATG	GCAGAGTCTCGCCATTATGATTC
*Prf1* NM_011073	AGCACAAGTTCGTGCCAGG	GCGTCTCTCATTAGGGAGTTTTT
*Gzmf* NM_010374	GCTGGGGGAGAACATCCATC	TGTCCTGTTTAGCCCATAGGT
*TGF-β* NM_009367	CTTCGACGTGACAGACGCT	GCAGGGGCAGTGTAAACTTATT
*Tnf-α* NM_009396	AGGAGGAGTCTGCGAAGAAGA	GGCAGTGGACCATCTAACTCG
*Ifn-γ* NM_008337.4	ATGAACGCTACACACTGCATC	CCATCCTTTTGCCAGTTCCTC

### Hematological Analyses

After euthanasia, blood was collected by cardiac puncture in EDTA (10.25 mg/mL) collection tubes and immediately processed. Analyses were performed on an automated hematology analyzer (BC-2800Vet, Mindray, USA) using mouse-specific algorithms and parameters.

### Flow Cytometry 

Tumor was dissected and filtered through a cell strainer (100 µm, Corning, USA) in PBS. Red blood cells (RBC) were lysed using ACK (Ammonium-Chloride-Potassium) RBC Lysing Buffer (0.15 M NH_4_Cl, 10.0 mM KHCO_3_, 0.1 mM Na_2_ EDTA), and the B16-F10 and immune infiltrating cells were kept in PBS. One million cells per well were stained in a round bottom 96 well plate using a two-step staining protocol. First, cells were stained with a live/dead dye (Fixable aqua 405 nm, Invitrogen, USA) at 4°C for 20 min, cells were washed, and 100 μL final volume of a solution containing surface antibodies diluted in staining buffer (1% fetal bovine serum, FBS, 1 mM EDTA, and 0.02% NaN_3_ in PBS) were added into each well. After 30 min at 4°C, the samples were washed (2X) and resuspended in staining buffer until acquisition. The following antibodies were used: PerCP-Cy5.5 Anti-Mouse CD80 (Clone 16-10A1 Cat no. 194722), APC-Cy7 anti-Mouse F4/80 (Clone BM8 Cat no. 123118), FITC Anti-Mouse CD206 (Clone C068C2 Cat no.141704), from Biolegend, USA, and PE-Cy7 anti-mouse CD86 (Clone GL1 Cat no. 560582) from BD, USA. Samples were assessed with a FACSCanto II cell analyzer (Becton Dickinson, USA) using DiVA 8 acquisition software and FlowJo 5 V10 (Becton Dickinson, USA) data analysis software.

### Cell Isolation and Culture

All experiments using live cells were performed with murine splenic CD8+ T cells isolated with the Mouse CD8+ T Cell Isolation Kit (MACS Miltenyi Biotech, USA). Following isolation, cells were resuspended at 1 × 10^6^ cells/mL in T cell culture medium: RPMI 1640 medium (Thermo Fisher, USA) containing 10% FBS, 1X Glutamax (Life Technologies, USA), 1 mM sodium pyruvate, 0.1% β-mercaptoethanol, and 100 U/mL penicillin/ 100 µg/mL streptomycin (Gibco, USA). Cells were kept on ice, counted in a hemocytometer, and evaluated for viability using Trypan Blue (Gibco, USA) and immediately analyzed in the Seahorse experiment.

### Seahorse High Resolution Live Cell Respirometry

The oxygen consumption rate **(**OCR) and the extracellular acidification rate (ECAR) were recorded using a Seahorse XFe96 Analyzer (Agilent, USA). CD8+ T cells were freshly isolated and resuspended in Agilent XF Assay Medium supplemented with 25 mM glucose, 1 mM sodium pyruvate, and 2 mM L-glutamine. Cells (0.2 × 10^6^ cells/well) were then plated on Seahorse assay plates coated with poly-D-lysine (Sigma Aldrich, USA) and let to rest at room temperature in the hood for 30 min. During the assay, cells were kept in the same medium and exposed to 1 μM oligomycin, 1.5 μM carbonyl cyanide p-trifluoromethoxyphenylhydrazone (FCCP), 100 nM rotenone and 1 μM antimycin A, purchased from Sigma-Aldrich, USA, as indicated in the figures. Alternatively, a mix of phorbol myristate acetate (5 ng/mL, PMA) and ionomycin (1 μM), purchased from Sigma-Aldrich, USA, were used in the injections during the experiments. For some of the experiments, the seahorse plate was also coated with 2 μg/mL mouse anti-CD3 (Clone 145-2C11 Cat no. 553058) and 10 μg/mL mouse anti-CD28 (Clone 37.51 Cat no. 553294), both from Becton, Dickinson, USA, for 24 h, and washed twice with PBS before plating the cells.

### CD8+ T Cell Purification and *In Vitro* Cytotoxic T Lymphocyte (CTL) Assay 

Splenocytes collected from *Trpa1* ^+/+^ and *Trpa1* ^-/-^ mice were stained with fluorochrome-conjugated antibodies FITC anti-mouse CD4 (Clone RM4-5 Cat no. 100509, Biolegend, USA), PER-CP anti-mouse F4/80 (Clone BM8 Cat no. 123126, Biolegend, USA), APC anti-mouse CD19 (Clone 1D3 Cat no. 550992, BD, USA), PE anti-mouse CD105 (Clone MJ7/18 Cat no. 12-1051-82, eBioscience, USA), and BV421 anti-mouse CD11c (Clone HL3 Cat no. 562783 BD, USA) and sorted to obtain a purified and enriched CD8+ T cell population using a FACS (FACS Aria II Cell sorter, BD Biosciences). Meanwhile, B16-F10 WT cells cultured in RPMI 1640 medium (Atena, Brazil) with 10% FBS, to be used as target cells, were stained with a cell tracking marker (1 µL of dye per 10^6^ cells, Cell TraceCellTrace™ Violet Cell Proliferation Kit, Invitrogen, USA) following the manufacturer’s instructions. For *in vitro* stimulation, sorted CD8+ T cells (effector cells) were co-cultured on plate-bound anti-CD3 (2 μg/mL) (Clone 145-2C11 Cat no. 553058) and mouse anti-CD28 (10 μg/mL) (Clone 37.51 Cat no. 553294) with B16-F10 stained cells in a ratio of 5 CD8+ cells to 1 B16-F10 cell, and incubated at 37°C with 5% CO_2_ for 4 h (28, 29). Finally, co-cultured cells were stained using the two-step protocol using the following dye and fluorescent antibody: live/dead dye (Fixable aqua 405 nm, Invitrogen, USA) and APC anti mouse CD8 (Clone 53-6.7 Cat no. MCD0805, Invitrogen, USA), according to the staining method previously described. Samples were assessed with a FACSCanto II cell analyzer (Becton Dickinson, USA) using DiVA 8 acquisition software and FlowJo 5 V10 (Becton Dickinson, USA) data analysis software.

### CD8 T RNA-Seq Data Analysis

Data were retrieved from the Gene Expression Omnibus [accession GSE141465 (30)] using GEOquery and Biobase Bioconductor R packages (http://www.bioconductor.org/). In Parrot’s study (30), CD8+ T cells were sorted from metastatic melanomas (n = 8) expanded and stimulated or not for 6 h with plate-bound anti-CD3 (1 µg/mL). Gene expression of paired unstimulated/stimulated T CD8 samples was quantified using Illumina HumanHT-12 V4.0 Expression Beadchip arrays. Data were processed using quantile normalization and log2-transformed. For analysis, we first grouped CD8+ T samples according to their expression of *Trpa1* in basal conditions (unstimulated). Next, we evaluated the impact of CD3 activation in low *Trpa1* and high *Trpa1* samples and computed the log2 (fold change) in gene expression for each group separately (log2FClow and log2FChigh). For gene set enrichment analysis (GSEA), we ranked genes by comparing their differential expression upon CD3 activation in high *Trpa1* vs. low *Trpa1* samples (log2FChigh - log2FClow). GSEA was performed using the desktop application v.4.0.3 (31) and the Reactome (32), KEGG (33) and HALLMARK databases (34). Enrichment scores (ES) were calculated based on a weighted Kolmogorov–Smirnov-like statistic and normalized (NES) to account for gene set size. p-values corresponding to each NES were calculated using 1,000 gene set permutations and corrected for multiple comparisons with the false discovery rate (FDR) procedure. Differences were considered statistically significant for adjusted p-values (FDR q) < 0.05.

### The Cancer Genome Atlas (TCGA) RNA-Seq Data Analysis

TCGA RNA-seq and clinical data from 473 melanomas (35) were downloaded from the UCSC XENA Browser (36). Data were generated using the Illumina HiSeq 2000 RNA sequencing platform and quantified with RSEM. Estimated counts were upper quartile normalized and log_2_(normalized counts + 1). Estimation of the abundance of different immune cell types was calculated with CIBERSORT and the LM22 reference signature matrix, using the absolute mode, B-mode batch correction, disabled quantile normalization, and 100 permutations (37). One sample presented a p > 0.05 and was removed from the analysis.

### Statistical Analysis

Body weight, food intake, and tumor volume were analyzed by Two-Way ANOVA followed by Bonferroni’s post-test. Hematological analysis, flow cytometry, and gene expression assays were analyzed by unpaired Student’s *t*-test. For Seahorse data analysis, the area under the curve was calculated and the data were analyzed with One-Way ANOVA followed by Tukey for the comparison of more than two groups or with unpaired Student’s *t*-test for two group comparison. In all scenarios, p < 0.05 was established to reject the null hypothesis. GraphPad Prism 7.0 was used for all statistical analyses (USA).

## Results

### Melanoma Progression Is Delayed in *Trpa1^-/-^* Mice

The modulation of immune cells by metabolism has become one of the hallmarks of immune function. Although presenting a clear role in metabolic regulation (22), the influence of TRPA1 channel family on immunometabolic alterations during cancer progression has not been fully elucidated. In order to fill this gap, we used a model in which *Trpa1* ^+/+^ and *Trpa1* ^-/-^ mice were inoculated with B16-F10 melanoma cells to understand CD8+ T activation and tumor progression.

**Figure 1:**
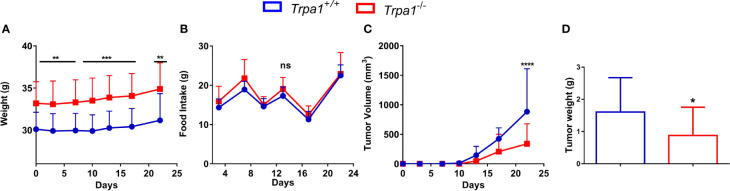
Evaluation of body weight, food intake, and tumor volume of *Trpa1* ^+/+^ or *Trpa1* ^-/-^ mice. Values are shown as mean (n = 13 for *Trpa1* ^+/+^ and n=18 for *Trpa1* ^-/-^) ± SD. All temporal analyses were carried out using Two-Way ANOVA followed by Bonferroni post-test. Tumor weight was calculated using unpaired Student’s *t-*test. **(A)** Animal weight; **(B)** Food intake; **(C)** Tumor volume; **(D)** Tumor weight. *p < 0.05; **p < 0.01; ***p < 0.001; ****p < 0.0001; ns, not significant, at each time point between genotypes.

We first evaluated the weight of tumor-bearing mice along the experiment and no temporal differences within each genotype were found. Interestingly, at all experimental time points, we found that *Trpa1* ^-/-^ animals were heavier than their wild-type counterparts (**Figure 1A**); however, such difference in weight was not associated with increased food intake (**Figure 1B**).

Despite the subtle differences in mice weight, a considerable difference in tumor growth could be observed. After inoculation with B16-F10 cells, the tumor was visible from the 13^th^ day onwards in both *Trpa1* ^+/+^ and *Trpa1* ^-/-^ mice. On the 17^th^ day, we found a reduction trend in tumor volume in *Trpa1* ^-/-^ compared to *Trpa1* ^+/+^ animals. On the 22^nd^ day, the difference became even more evident and statistically significant, i.e., the tumor volume and weight were significantly reduced in *Trpa1* ^-/-^ mice compared to *Trpa1* ^+/+^ animals (**Figures 1C, D**). In order to verify if these striking differences in melanoma progression could be related to a difference in immune surveillance, we evaluated the circulating pool of immune cells in these two genotypes in the absence or presence of the tumor.

### The Pool of Circulating Lymphocytes Is Higher in *Trpa1^-/-^* Mice

**Figure 2:**
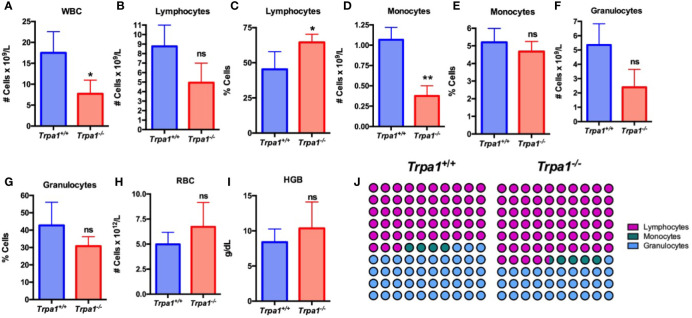
Blood analysis of *Trpa1* ^+/+^ or *Trpa1* ^-/-^ mice on the 22^nd^ day after B16-F10 cell inoculation. **(A)** White blood cells; **(B, C)** Absolute number and percentage of lymphocytes; **(D, E)** Absolute number and percentage of monocytes; **(F, G)** Absolute number and percentage of granulocytes; **(H)** Red blood cells; **(I)** Hemoglobin; **(J)** Representative percentage of lymphocytes, granulocytes, and monocytes. Values are shown as mean (n = 3 for *Trpa1* ^+/+^ and n=4 for *Trpa1* ^-/-^) ± SD. *p < 0.05; **p < 0.01; ns, not significant. Statistical analyses were performed by Student’s *t*-test between the genotypes.

To determine the relative levels of circulating immune cells in the *Trpa1^+/+^* and *Trpa1^-/-^* mice, we used an automated hemocytometer to evaluate the main cellular components of the circulating blood after tumor inoculation (**Figures 2A–J**). The total number of circulating white blood cells (WBC) was lower in the mutant mice as compared to the wild type animals, suggesting a higher level of recruitment to the tumor microenvironment (**Figure 2A**). The main striking difference between these groups was not associated with total number (**Figure 2B**), but with the overall higher percentage of circulating lymphocytes (**Figure 2C**) and smaller absolute number (**Figure 2D**), but not frequency (**Figure 2E**), of monocytes in *Trpa1^-/-^* mice. No difference of granulocytes, red blood cells (RBC), and hemoglobin was found between *Trpa1^-/-^* and wild type mice (**Figures 2F–I**). The differences (**Figures 2B–G**) became even more evident when the relative abundance of lymphocytes, monocytes, and granulocytes was plotted in a percentage image, as observed in **Figure 2J**.

Interestingly, healthy animals that were not submitted to PBS or melanoma cell inoculation, did not show any differences in lymphocyte, monocyte, granulocyte, RBC absolute number and percentage, and hemoglobin levels between the genotypes (**Supplementary Figure 1**).

Since the animals of the cohort were not injected with PBS and did not suffer the same experimental manipulation of the tumor-inoculated mice, we did not compare tumor inoculated with non-PBS injected animals. Our data, therefore, suggest that the differences seen in tumor inoculated mice are due to the presence of tumor cells.

### There Are No Significant Differences in Pro- and Anti-Inflammatory Populations of Tumor Associated Macrophages Between *Trpa1^+/+^* and *Trpa1^-/-^* Mice

As one of the most well-established mechanisms of tumor development is the growth benefits generated by tumor-associated macrophages (TAM), and knowing that these cells can originate from monocyte recruitment and differentiation, we sought to investigate if there were differences in the relative abundance of TAMs between *Trpa1^+/+^* and *Trpa1^-/-^* mice.

**Figure 3:**
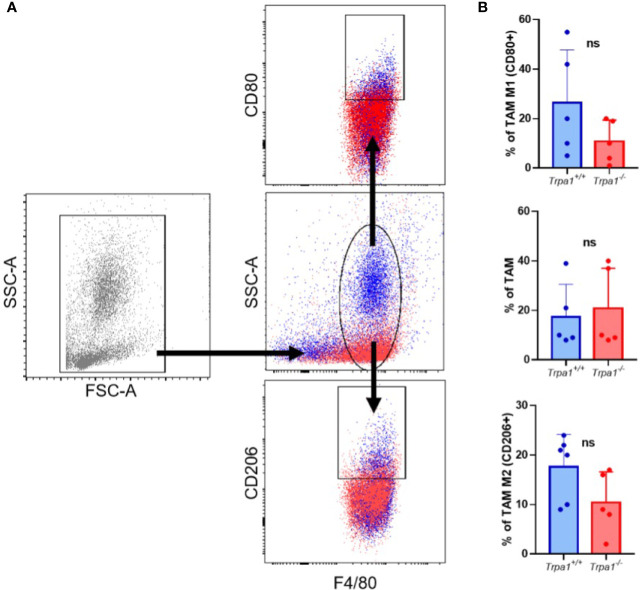
Evaluation of tumor-associated macrophages (TAM) from *Trpa1^+/+^* or *Trpa1^-/-^* mice. **(A)** Gating strategy for the definition of TAM populations; **(B)** Analysis of frequency of TAM in tumors. Subpopulations of M1 and M2 TAM were analyzed. Values are presented as the mean (n = 5) ± SD of the frequency (%) in each group. ns, not significant.

To evaluate TAM frequency, present in tumor microenvironment, we dissociated the tumor mass and stroma on the 22^nd^ day after inoculation and determine the main population expressing the surface marker F4/80 (total macrophages) by flow cytometry, observing no significant differences between *Trpa1* ^+/+^ and *Trpa1* ^-/-^ inoculated mice (**Figures 3A, B**). Moreover, when this population was further subdivided into CD80+ pro-inflammatory CD206+ resolving/anti-inflammatory macrophages, we observed a slight increase in some *Trpa1^+/+^* mice, but this was also not significant (**Figures 3A, B**).

Since differences in TAM populations did not seem to account for the delayed tumor progression in *Trpa1^-/-^* mice, we further investigated if this might be due to the difference observed in circulating lymphocytes.

### There Are Striking Differences on CD8+ T Lymphocyte Abundance and Activation in Tumor Microenvironment Between *Trpa1^+/+^* and *Trpa1^-/-^* Mice

**Figure 4:**
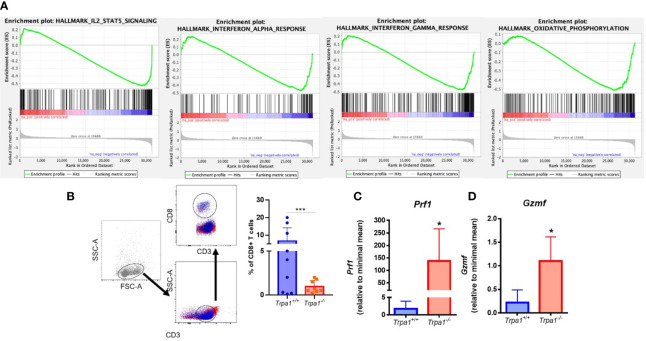
**(A)** Analysis of transcriptome data of T CD8+ cells sorted from human patient tumors. We used GSEA to compare the changes in gene expression induced by CD3 activation in *TRPA1* low and high cells. FDR-adjusted p values < 0.05 were considered statistically significant. IL-2 and Stat1 NES: -2,05246 and FDR: 1,00e-06; Oxidative phosphorylation NES: -1,9378266 and FDR 1,00e-06; IF gamma response NES: -1,8219428 and FDR 4,22e-04; IF alpha response NES: -1,6389698 and FDR 3,92e-03; **(B)** Gating strategy for the definition of CD8+ T cell populations and analysis of frequency of infiltrating CD8 T cells in tumors from *Trpa1^+/+^*or *Trpa1^-/-^* mice. **(C, D)** Bulk tumor mRNA isolation and qRT-PCR analysis 22 days after subcutaneous inoculation of B16-F10 cell inoculation; **(C)** Perforin mRNA relative levels; **(D)** Granzyme mRNA relative levels. Statistical analyses were performed by Student’s *t*-test. Values are presented as the mean (n = 9) ± SD of the frequency (%) in each group. *p < 0.05; ***p < 0.001.

We initially analyzed human T lymphocytes using a public available transcriptome dataset of the tumor infiltrating CD8+ T cells isolated from patients with metastatic melanoma, which were obtained through cell sorting, expanded *in vitro*, and stimulated with CD3+ for 6 h (30). CD8+ T cells were stratified into high and low *TRPA1* expression and gene enrichment analysis was performed using KEGG, Reactome, and GSEA datasets. Interestingly, we observed a negative correlation of *TRPA1* transcripts with several immune system-related datasets such as IL-2, IL-6, inflammatory response, cytokine and cytokine receptor interaction, interleukin signaling, and several others (**Figure 4A**). Therefore, CD8+ T cells displaying lower levels of *TRPA1* correlate with several immune system pathways associated with increased immune system activation. Furthermore, using the dataset of cutaneous melanoma from the TCGA, we implemented the Cibersoft algorithm, which is used to estimate the frequency of different immune system cells in the tumor bulk. In *TRPA1* low primary melanoma increased frequency of activated natural killer, resting dendritic, and eosinophils were found compared to *TRPA1* high tumors (**Supplementary Figure 2A**). However, in metastatic melanoma differential frequency of immune system cells were less prominent. Only CD4+ naïve T and mast resting cells were in higher and lower frequency, respectively, in *TRPA1* low metastatic tumors compared to *TRPA1* high ones (**Supplementary Figure 2B**). These data suggest that *TRPA1* likely plays a more complex role in tumor microenvironment in melanoma cancer.

We then analyzed the relative abundance of T lymphocytes in the tumor microenvironment. Using flow cytometry, we found out that the frequencies of CD3+CD4+ lymphocytes, B cells, and natural killers (NKs) were not significantly different between the two genotypes in both spleen and tumor stroma (**Supplementary Figures 3** and **4**). A distinct pattern was observed for CD3+CD8+ lymphocytes (CD8+ T), that presented significantly lower levels in the *Trpa1^-/-^* group (**Figure 4B**). These cells display important effector functions after recognizing dysfunctional somatic cells such as tumor cells, and release the cytotoxins perforin, granzyme, and granulysin. It is important to stress that after activation the CD8+ T cytotoxic cells also undergo programmed cell death due to their intrinsic effector function, so a lower frequency of this population, as observed in the *Trpa1^-/-^* group, actually suggests that these cells are more active in the tumor site in these animals.

Through the action of perforin, granzyme enter the target cell and its serine protease activity triggers the caspase cascade leading to apoptosis. The relative levels of both perforin (**Figure 4C**, *Prf1*) and granzyme (**Figure 4D**, *Gsmf*) observed in the tumor stroma were highly upregulated in the *Trpa1^-/-^* group, supporting the fact that in this group the CD8+ T cytotoxic effector function was more prevalent that in the wild type group. We also found increased expression of *Il-1β, Il-6*, and *Ifn- γ* in tumor stroma from *Trpa1^-/-^* group compared to wild type animals (**Supplementary Figure 5**).

Recently it has been reported that TRPA1 can modulate the metabolism in mammals (14, 38) and this became clearer as one of the GSEA pathways associated with TRPA1 relates to oxidative metabolism in humans (**Figure 4A**). Knowing that this is a regulatory hub for cytotoxic lymphocyte response during tumor onset and development, we decided to evaluate the metabolism of these cells in *in vitro* experiments.

### TRPA1 Modulates Both Glycolysis and the Oxidative Metabolism of CD8+ Cytotoxic T Lymphocytes Upon Stimulus

To investigate the possible role of TRPA1 channel in modulating the metabolic phenotype displayed by CD8+ T lymphocytes, we isolated fresh unstimulated cells from the spleen of the *Trpa1^-/-^* and wild type animals and assessed their extracellular acidification rate (ECAR) and oxygen consumption rate (OCR) as a proxy of glycolysis and oxidative metabolism, respectively.

**Figure 5:**
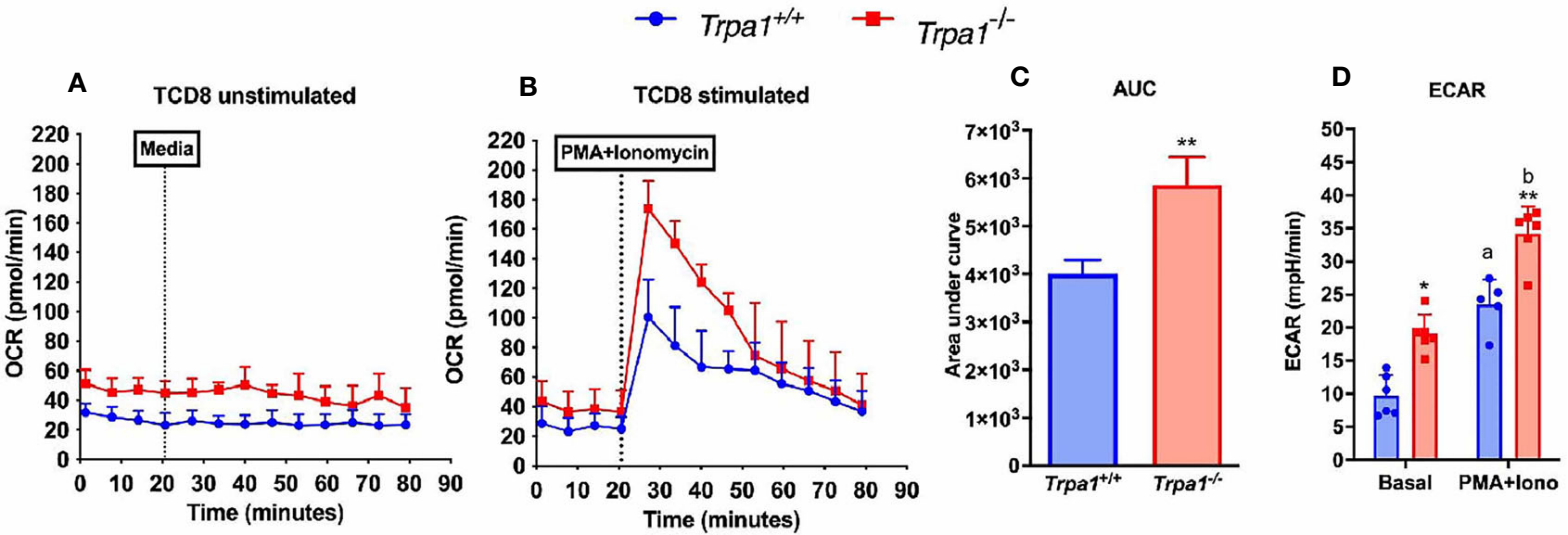
Metabolic parameters from spleen-derived *Trpa1* ^+/+^ or *Trpa1* ^-/-^ T CD8 lymphocytes. **(A)** Basal unstimulated levels of oxygen consumption rate (OCR) of freshly isolated T CD8 cells; **(B)** OCR levels of T CD8 exposed to a cocktail with ionomycin and PMA compared to the basal rate; **(C)** Area under the curves of B; **(D)** Extracellular acidification rates (ECAR) for both non-stimulated and stimulated cells from the two groups. Values are presented as the mean (n = 8) ± SD of each group. Statistical analyses were performed by Student’s *t*-test in C and by One Way ANOVA followed by Tukey in **(D)** Each well contained 100,000 cells. *p < 0.05; **p < 0.01 between genotypes. Difference between the different conditions within the same genotype is represented by letters a ≠ b, p < 0.05.

When unstimulated CD8+ T lymphocytes from *Trpa1^-/-^* and *Trpa1^+/+^* mice were compared, we observed a slight increase in the resting state of OCR, although not statistically significant, in the overall number of the knockout cells (**Figure 5A**). This suggests that at the resting/non activated state these groups present comparable levels of oxidative metabolism. A remarkable difference can be observed when these two groups were stimulated with a cocktail of ionomycin and phorbol ester, PMA, simulating the signaling transduction elicited in the immunological synapsis with an antigen-presenting cell. With activating stimuli, the *Trpa1^-/-^* group displayed almost immediately a significant increase in their oxygen consumption (**Figure 5B**) that was significantly higher than the one from the *Trpa1^+/+^* group, as can be seen in the quantification of the area under the curves (**Figure 5C**). The increased level of oxidative metabolism could mean a better metabolic activation capacity towards consuming energetic substrates in the mitochondria along with a higher rate of ATP production.

Moreover, upon determining the lactate production using the extracellular acidification rate as a proxy for glycolysis, we observed that in the non-stimulated condition the *Trpa1^-/-^* group already displayed significantly higher ECAR levels than the wild type animals, and upon stimulation this was even more evident (**Figure 5D**), suggesting that these cells are capable of a more robust bioenergetic shift towards allocating energetic reserves during activation.

Based on these data, we went on to fully characterize the resting and stimulated metabolic states in these cells using a series of drugs commonly used to evaluate the behavior of the mitochondrial ATP synthase and electron transport chain and the associated parameters.

### The Lack of TRPA1 Dramatically Increases the Maximal Mitochondrial Respiration of CD8+ T Cytotoxic Lymphocytes Upon Stimulus Impacting Spare Respiratory Capacity Along With Glycolysis

**Figure 6:**
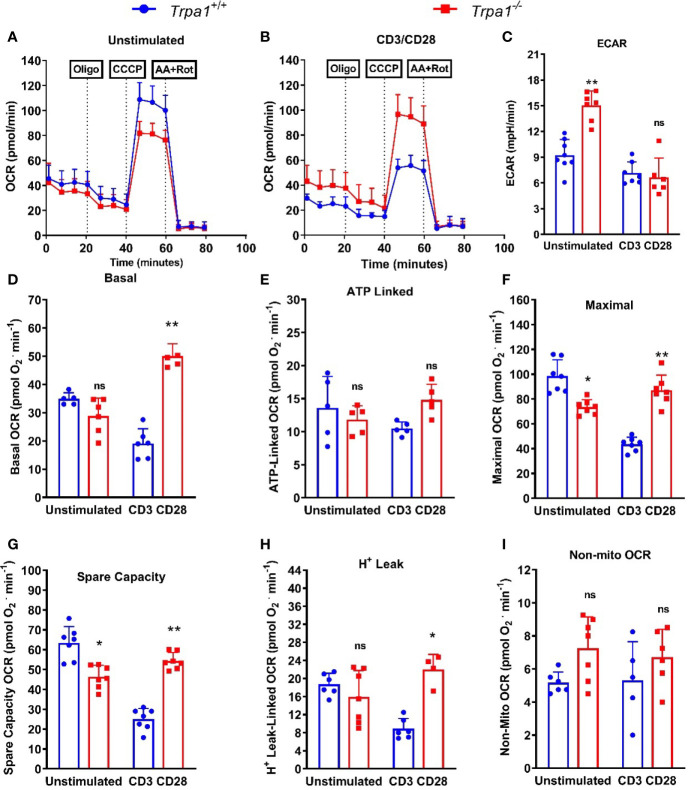
Mitochondrial metabolic evaluation from spleen-derived *Trpa1^+/+^* or *Trpa1^-/-^* T CD8 lymphocytes under CD3/CD28 activation. **(A)** Traces obtained from unstimulated` *Trpa1^+/+^* or *Trpa1^-/-^* T CD8 cells after injections of oligomycin (ATP synthase inhibitor), CCCP (uncoupler) and antimycin A plus rotenone (Complex III and I inhibitors); **(B)** Same as in A but cells were stimulated for 30 min using immobilized CD3/CD28; **(C)** Extracellular acidification rate (ECAR) from unstimulated and stimulated cells; **(D)** Basal respiration; **(E)** ATP-linked oxygen consumption rate; **(F)** Maximal oxygen consumption rate; **(G)** Spare capacity (difference between maximal and basal respiration); **(H)** Proton-leak linked oxygen consumption rate; **(I)** Non-mitochondrial respiration (this part is subtracted from all the other respiration rates shown in **D–H**). Values are presented as the mean (n = 8) ± SD of each group. *p < 0.05; **p < 0.01. One-Way ANOVA analysis followed by Tukey was performed to evaluate differences between conditions and genotypes.

To examine whether there were differences in the metabolism of CD8+ T lymphocytes from *Trpa1^-/-^* and *Trpa1^+/+^* mice, we isolated and immobilized these cells for intact cell respiration using the Seahorse technology. We observed (**Figure 6A**) that the oxygen consumption rates between these two groups did not display significant differences when these cells were in the non-stimulated basal condition. This result contrasts to what was observed after a 30 min-long stimulation with anti-CD3+ and CD28+ antibodies (**Figure 6B**). The first intriguing observation is that only non-stimulated CD8+ T cells from *Trpa1^-/-^* mice presented increased levels of ECAR indicating that this group exhibits a predisposition to higher glycolytic rates (**Figure 6C**). As this has been directly associated with the cytotoxic function of these cells *in vitro*, it is interesting to speculate that maybe this poises these cells with an advantage in the capacity to be activated when dealing with the tumor progression.

We next examined the basal respiration, or the consumption of oxygen by the cell mitochondrial network under basal condition using only culture medium. Under this condition the basal respiration of *Trpa1^-/-^* cells was slightly lower than of their *Trpa1^+/+^* counterparts, but when activated these cells go on to fully display increased basal respiration. These data suggest that their oxidative metabolism is upregulated most probably to generate more ATP and or precursors necessary for their killing activity (**Figure 6D**).

To further characterize the metabolic response in these two groups, we used a treatment comprising the injection of oligomycin, that inhibits the ATP synthase, and we could observe that there were no significant differences in the ATP-linked oxygen consumption between the two groups (**Figure 6E**), suggesting that the majority of the ATP that fuels the difference response of these CD8+ T cells is most likely derived from the substrate level generation found in glycolysis. Next, we characterized the mitochondrial maximal and spare respiratory capacity using an uncoupler drug (CCCP) that dissociates the flux of electrons from the oxygen consumption in the mitochondria leading to maximal rates in the OCR. Our first observation is that both maximal, as well as spare respiratory capacity, presented a similar behavior (**Figures 6F, G**, respectively). The levels of maximal respiration in non-activated *Trpa1^-/-^* CD8+ T cells were slightly, but significantly, lower than of their wildtype counterparts but this pattern inverted after CD3/CD28 co-stimulation, as the knockout group presented a significantly higher maximal oxygen consumption (**Figure 6F**). This, coupled with higher levels of glycolysis displayed by these cells, allows for a substantial glycolytic shift without losing mitochondrial activity which is advantageous *per se*. The same can be observed for the spare respiratory capacity (**Figure 6G**), which in general correlates with better physiological responses in cells exposed to metabolic challenges. The same could be observed for the proton leak (**Figure 6H**) that relates to their uncoupled state. These data suggest that *Trpa1^-/-^* CD8+ T cells would be in better conditions to modulate their metabolism when facing the activation stimuli. We also evaluated the levels of non-mitochondrial respiration by adding inhibitors of the mitochondrial complexes I and III (rotenone and antimycin A, respectively), but could not observe any significant differences in this activity (**Figure 6I**).

Taken altogether, these data suggest that *Trpa1^-/-^* CD8+ T lymphocytes can modulate their metabolism in a more prominent way when activated. Subsequently, we investigated whether this metabolic plasticity would entail these cells with a more proficient killing activity.

### TRPA1 Impacts the Cytotoxic Response Proficiency of CD8+ T Lymphocytes

**Figure 7:**
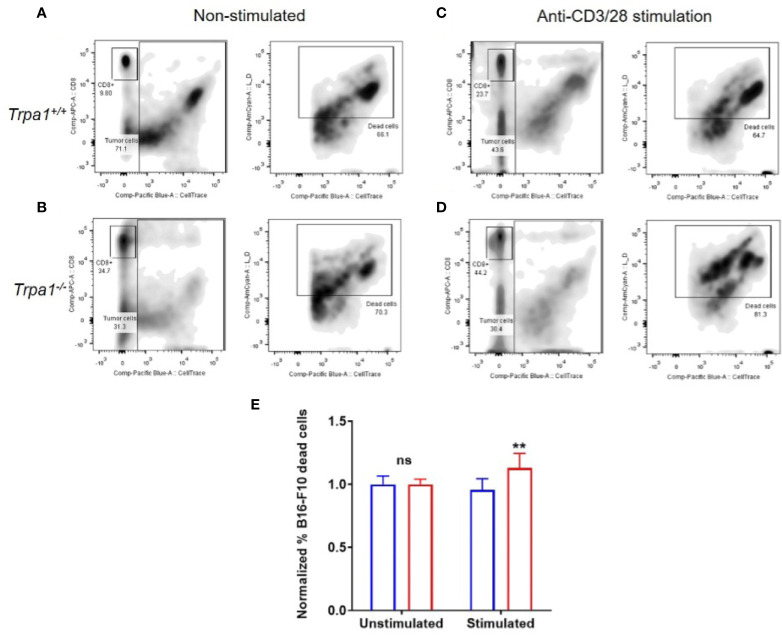
Killing capacity of CD8^+^ cells from *Trpa1* ^+/+^ and *Trpa*1^-/-^ mice co-cultured with B16-F10 cells. A and B) Representative assessment of B16-F10 cell killing by CD8^+^ T cells from *Trpa1^+/+^* **(A)** and *Trpa1^-/-^* **(B)** mice under non stimulated conditions. C and D) Representative assessment of B16-F10 cell killing by CD8^+^ T cells from *Trpa1* ^+/+^ **(C)** and *Trpa1* ^-/-^ **(D)** mice under CD3/CD28 stimulation. **(E)** Normalized frequency of B16-F10 cell death by CD8^+^ T cells from *Trpa1* ^+/+^ and *Trpa*1^-/-^ mice; values are presented as the mean (n = 3 and 6 for unstimulated and stimulated group, respectively) ± SD. Statistical analyses were performed by Student’s *t*-test. **p < 0.01, ns, not significant.

To evaluate the metabolic plasticity and efficiency of these cells to undergo an increased oxidative response while also ramping up the glycolysis, we sorted CD8+ T cells from the spleen of *Trpa1^+/+^* and *Trpa1^-/-^* mice, stimulated these cells with immobilized CD3+ and CD28+ antibodies for 30 min and then exposed them to B16-F10 melanoma cells in a 5:1 ratio (5 tumor cells to 1 CD8+ T cell). Four hours later, the number of dead tumor cells was quantified by using the loss of membrane integrity and covalently protein binding flow cytometry coupled with discrimination and exclusion of the lymphocytes through CD8+ T staining (**Figures 7A–D**).

Using this approach, we did not find any difference in cytotoxic activity of CD8+ T cells from either *Trpa1^+/+^* or *Trpa1^-/-^* mice in the unstimulated scenario; however, upon stimulation with CD3/CD28 antibodies, there was an increase in cytotoxic activity of *Trpa1^-/-^* CD8+ T cells compared to wild type cells (**Figure 7E**). Collectively these data show that the increased metabolic capacity displayed by *Trpa1^-/^* ^-^ CD8+ T cells directly translates into a better cytotoxic performance.

## Discussion

Tumors promote a major disturbance to tissue homeostasis: They create energetic and substrate demanding environments that impact on metabolism and function of the stroma and infiltrating immune cells. Nutrient competition is at bay as the unrestrained cell growth seen in cancer is often supported by aerobic glycolysis, the same metabolic pathway needed to fuel optimal effector functions in many immune cells (18). The demand for nutrients, essential metabolites, and oxygen imposed by proliferative cancer cells creates harsh environmental conditions in which immune cells must navigate and adapt. How tumor and immune cells share or compete for resources in this microenvironment, and how such relationships regulate antitumor immunity are pressing questions to address.

At the forefront of these mechanisms, how immune cell metabolism, and thus immune cell function, is altered by the tumor microenvironment, is a question of utmost interest and importance. To address this question, we evaluated the participation of the TRPA1 channel in the immune response against melanoma tumor progression in a model of murine melanoma. Recently, Antoniazzi and colleagues (39) showed that tumor growth, through injection of B16-F10 cells in the mouse paw, is not different between *Trpa1* ^+/+^ and *Trpa1* ^-/-^ genotypes, which contrasts with our results. However, we highlight some methodological differences that impair the comparison between that study and our data presented here: 1) In (39) a *Trpa1* ^-/-^ mice in C57 background was used while we used knockouts in B6;129 background along with its appropriate control; 2) we injected 2x10^6^ B16-F10 cells in the right flank while Antoniazzi and coworkers (39) inoculated 2x10^5^ cells in the paw. Nonetheless, using *Trpa1* ^+ / +^ and *Trpa1* ^- / -^ animals, we followed tumor progression using B16-F10 cells and assessed isolated CD8 + T cells for respiratory and cytotoxic function with an in depth look on how immunometabolism contributes to cell function during cancer progression.

Cytotoxic CD8+ T lymphocytes (CTL) play an essential role in providing effective antigen-specific immunity against tumors. CTL recognizes tumor-associated antigens presented on major histocompatibility complex class I (MHCI) by their expressed T cell receptor (TCR) and destroys target tumor cells through different mechanisms. This includes release of granules containing perforin and granzymes and inducing FasL-mediated apoptosis. However, to achieve long-lasting anti-tumor immunity, it is necessary to establish memory CD8+ T cell responses (40, 41). CTL populations have been classified by several surface markers and distinguished by their functions and residency, along with their effector cytokine production. Naïve CTL cells possess strong proliferative potential after antigen stimulation and resist terminal differentiation and exhaustion when compared with memory T cells which can rapidly produce multiple functional molecules after restimulation to control the tumor progression (42, 43).

While naive CD8+ T cells have relatively low energetic requirements, effector T cells present an increased demand for energy and biosynthetic precursors to support proliferation and effector function. Signaling in T cells through the TCR receptor and co-stimulatory molecules (such as CD28 and cytokine receptors) leads to the activation of immunological pathways which are accompanied by a profound alteration in the cellular metabolism to support their proliferation and effector function (44).

The TRPA family contains only one member, TRPA1, in vertebrates. This ion channel is best known in sensory neurons as a sensor for environmental irritants, inflammatory pain, and itching, but it has a diverse tissue distribution and plays different roles in a variety of non-neuronal cells (45). The constitutive expression of TRPA1 mRNA and protein in mouse and human primary CD4+ T cells controls CD4+ T cell activation and pro-inflammatory responses in models of colitis (45). In a recent study, Sahoo and colleagues reported TRPA1 expression in murine and human T CD3+ cells. Interestingly, TRPA1 inhibition prevents CD25 and CD69 expression and tumor necrosis factor (TNF), interferon γ (IFN-γ), and interleukin 2 (IL-2) secretion by stimulated T cells (46). Although not providing the data in the manuscript, the authors stated that inhibition of TRPA1 prevented T cell activation of CD4+ and CD8+ (46). Such findings seem to be in contradiction to our data since the lack of TRPA1 in our experimental model augmented T cell activity with a metabolic shift and increased cytotoxicity activity. However, it should be stressed that the mentioned study used BALB/c mice while we used B6;129 animals and no cytotoxicity assay was performed in that study (46). In addition, the lack of TRPA1 could lead to a compensatory mechanism, which would ultimately lead to increased T cell response. As TRP channels, including TRPA1, are known to form heterodimers especially with TRPV1 (47), a putative compensation between TRP channels could affect our results and prompts for future investigation.

Research over the past decade shows the critical role of TRPA1 as a sensor of inflammation throughout the body [as summarized in (11)]. In this review the authors draw attention to the fact that there is an increasing appreciation of the role that chronic inflammation plays in tumorigenesis and of the presence of inflammation in the tumor microenvironment. A possible unknown role of TRPA1 that may contribute to the pathogenesis of cancer and other inflammatory diseases is its role in modulating the metabolism of CD8+ T cells. If true, this could serve as the mechanistic explanation as to why we observed such a great degree of delay in tumor progression in *Trpa1* ^- / -^ mice inoculated with melanoma cells. Moreover, it could also explain why the relative composition of the circulating and local immune cells differ so drastically in the presence and absence of this channel. And finally, more importantly, it could suggest that *Trpa1* ^+/+^ and *Trpa1* ^- /-^ CD8+ T cells would express a completely different metabolic landscape, which was one of our objectives.

Initially described as a “Warburg-like” effect (48), current knowledge states that the activation of T cells does not lead to a complete switch from mitochondrial respiration toward aerobic glycolysis. Indeed, mitochondrial oxidation plays an important role in CD8+ T cell activation, as evidenced by the deleterious effects that inhibiting mitochondrial function has on T cell differentiation (44).

During cancer progression both tumor and immune cells are in active competition for nutritional resources due to limited availability of glucose, amino acids, fatty acids and oxygen within the tumor microenvironment. Thus, a better glycolytic and oxidative metabolic capacity displayed by the *Trpa1* ^-/-^ CD8+ T lymphocytes could translate into a more effective clonal expansion capacity of CD8+ effector T cells, since they both rely on the coexistence of robust bioenergetic catabolism and concomitant anabolism (49).

*In vitro* activated mouse CD8+ T cells exhibit both higher ECAR and OCR compared with naïve T cells, indicating that both glycolysis and oxidative phosphorylation, respectively, can be engaged to meet important increased metabolic demands upon T cell activation (50). Moreover, mitochondrial oxidation is a hallmark of CD8+ T memory cell development (51). In an *in vitro* study, van der Windt and colleagues (51) have shown that memory CD8+ T cells possessed substantial mitochondrial spare respiratory capacity. They found that interleukin-15 (IL-15), a cytokine critical for CD8+ memory T cells, regulates oxidative metabolism by promoting mitochondrial biogenesis and expression of carnitine palmitoyl transferase (CPT1a), a metabolic enzyme that controls the rate-limiting step to mitochondrial fatty acid oxidation.

The development and optimal function of anticancer memory CD8+ T cells rely on efficient fatty acid oxidation and can be boosted by inhibiting glycolysis, as at least in part, this effect stems from metabolic reprogramming involving increased oxidative phosphorylation (49). This resonates with our finding showing increased oxygen consumption rates in activated *Trpa1* ^-/-^ CD8+ T lymphocytes.

Another interesting finding supporting our data has been recently published pertaining the roles of interleukin 2 (IL-2) and IL-21 in T CD8 cells (52), cytokines that shape CD8+ T cell differentiation. IL-2 drives terminal differentiation, generating cells that are poorly effective against tumors, while IL-21 promotes stem cell memory T cells. The authors describe that the exposure to IL-2 promoted effector-like metabolism and aerobic glycolysis, robustly inducing lactate dehydrogenase (LDH) and lactate production, whereas IL-21 elicited a state dependent on mitochondrial oxidative phosphorylation. Even more interesting, the transient inhibition of LDH in these cells enhanced the generation of memory cells capable of triggering robust antitumor responses after adoptive transfer, thus showing how important the oxidative metabolism for effector function is. Accordingly, we found that *Trpa1* ^-/-^ CD8+ T cells were more effective in killing tumor cells *in vitro* and *in vivo*, which highlights the importance of these immunometabolic modulatory changes exerted to immune cell function. Corroborating our experimental data, we discovered that *TRPA1* is negatively correlated with several immunological related pathways in CD8+ T cells isolated from metastatic melanoma patients. Thus, reduced *TRPA1* expression in CD8+ T cells is associated with increased immune system activation, as it has been demonstrated in our *in vitro* and *in vivo* mouse experiments.

Finally, it is known that tumor-infiltrating CD8+ T cells undergo metabolic exhaustion in the nutrient and oxygen-deprived tumor microenvironment (48). Thus, reprograming CD8+ T cell metabolism may provide important therapeutic strategies for cancer treatment. Indeed, the adoptive transfer of memory CD8+ T cells with sustained metabolic fitness may yield better antitumor protection in both mouse model and the clinic (50). Here we show that *Trpa1* ^-/-^ CD8+ T cells display an impressive capacity of metabolic shift with enhanced killing activity that slows down tumor progression *in vivo.* It should be mentioned that our study used one melanoma cell line (B16-F10). It is unclear whether the described events in this study would also happen in different murine and human melanoma cell lines, which is a matter of further investigation. It is an open question the role of TRPA1 channel in CD8+ T cells in a metastatic cancer model, and further studies are necessary. Moreover, we decided to study male mice to avoid confounding factors of the female gender such as estrous cycle-dependent changes in tumor development. Therefore, our findings cannot be extrapolated to females, which may be another limitation of our study. Nevertheless, our data have opened a new venue to explore this ion channel as a target for immune cell-based therapies. We hope that our findings spark a deeper investigation of TRPA1 and other TRP channels in cancer development. Within this line, our data suggest that TRPA1 could be an important player in modulating T-dependent responses in the tumor microenviroment. The modulatory role of TRPA1 channel may also affect other types of immune system and healthy cells in the tumor microenviroment, and therefore, may result in exciting putative pharmacological targets in melanoma treatment.

## Data Availability Statement

The original contributions presented in the study are included in the article/**Supplementary Material**. Further inquiries can be directed to the corresponding author.

## Ethics Statement

All experimental procedures were performed according to Brazilian legislation approved by the Committee for Animal Use (CEUA IB/USP, number 255/2016, 14th of June 2016).

## Author Contributions

MF, LA, OD-A, NC, and AC conceptualized the study and the original hypothesis. LA together with MM collected data for mice weight, food intake, and tumor volume. MF, LA, and OD-A performed experiments, analyzed the data. GK researched the bioinformatic data together with LA. LA, MF, and OD-A wrote together the first draft of the manuscript. AC and NC supervised the study, ensuring rigorous data quality control, and contributed to the discussion, and critically revised the manuscript. All authors contributed to the article and approved the submitted version.

## Funding

AC’s lab is supported by the Sao Paulo Research Foundation (FAPESP, 2017/24615-5, and 2018/14728-0) and by the National Council of Technological and Scientific Development (CNPq 303078/2019-7). NC’s lab is supported by FAPESP (2017/05264-7). MM is a Young Investigator of FAPESP (2017/26651-9). LA and OD-A are fellows of FAPESP (2018/16511-8 and 2017/16711-4, respectively). MF is supported by a fellowship of the Pew Latin American Fellow Program in the Biomedical Sciences from Pew Charitable Trusts.

## Acknowledgments

The authors thank Renata Alves dos Santos for providing excellent care of the mice.

## Conflict of Interest

The authors declare that the research was conducted in the absence of any commercial or financial relationships that could be construed as a potential conflict of interest.
