# Supplementary material for: An Immunometabolic Shift Modulates Cytotoxic Lymphocyte Activation During Melanoma Progression in TRPA1 Channel Null Mice
Source: Front Oncol. 2021 May 10;11:667715. doi: 10.3389/fonc.2021.667715 (PMC8141816; doi:10.3389/fonc.2021.667715)
Supplement: Supplementary file 1 [file DataSheet_1.pdf]

Supplementary Figures

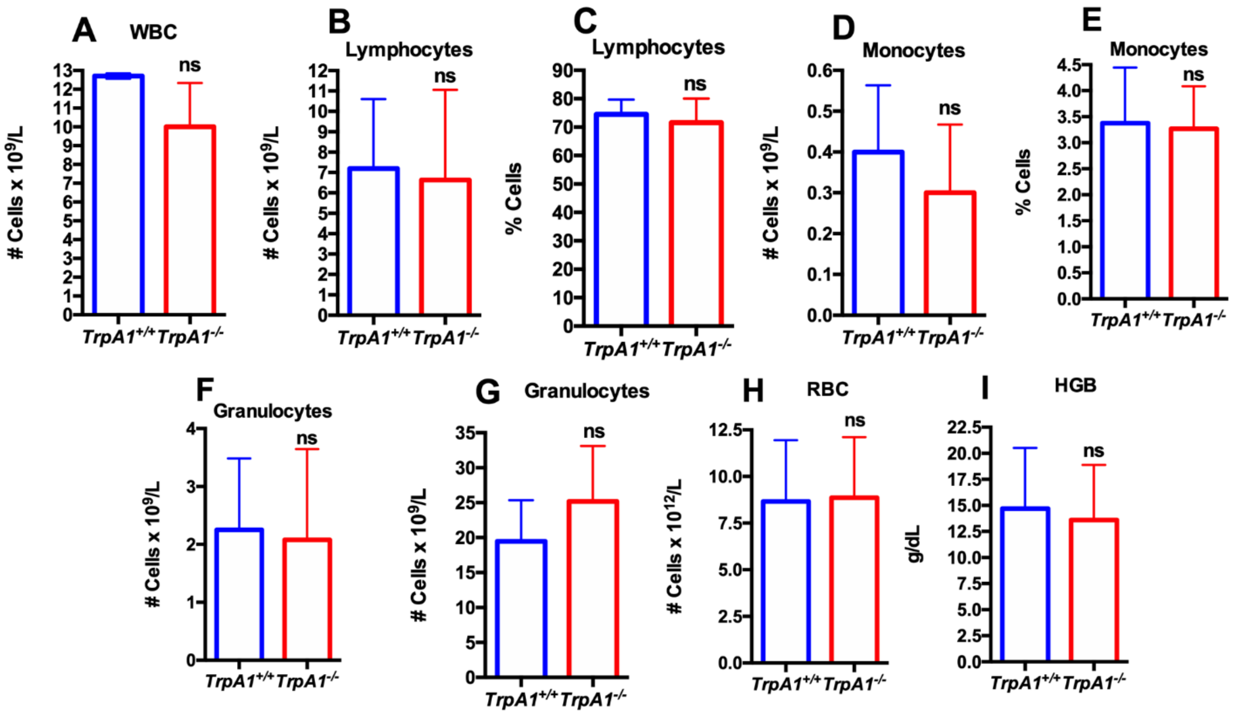

Supplementary Figure 1. Blood analysis of healthy, non-tumor inoculated, *Trpa1*$^{+/+}$ or *Trpa1*$^{-/-}$ mice kept in the same conditions as the tumor-inoculated ones. (A) White blood cells; (B – C) Absolute number and percentage of lymphocytes; (D – E) Absolute number and percentage of monocytes; (F – G) Absolute number and percentage of granulocytes; (H) Red blood cells; (I) Hemoglobin. Values are shown as mean (n = 3 – 6) ± SD. Statistical analyses were performed by Student's *t*-test test between the genotypes. ns=not significant.

A) Primary melanoma

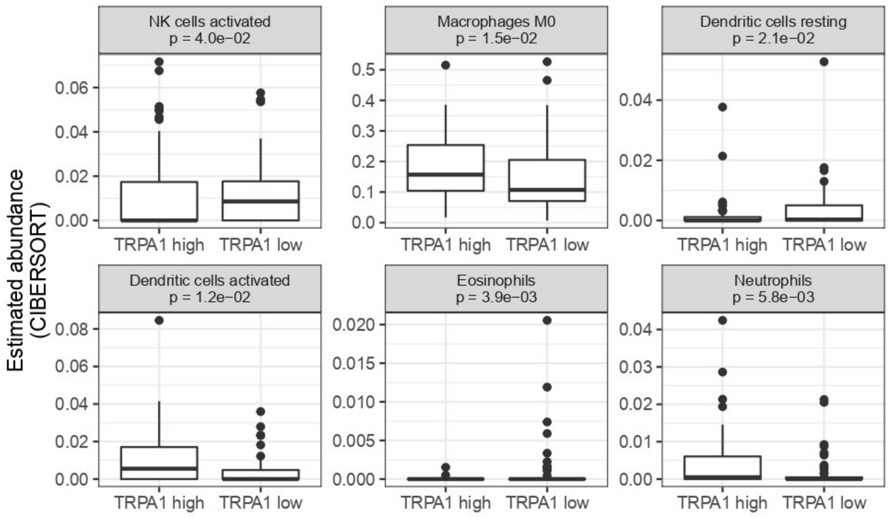

B) Metastatic melanoma

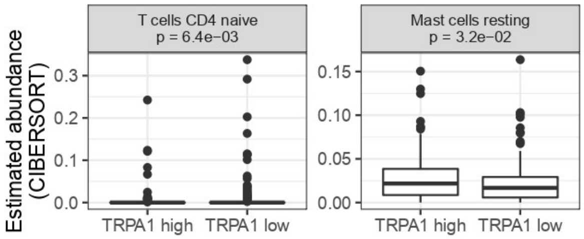

Supplementary Figure 2. Estimate frequency of immune system using Cibersoft algorithm in human primary (A) and metastatic melanoma (B) from TCGA database. Tumors were classified in *TRPA1* high and low expressing tumors and different populations of immune cells are represented with their respective p value.

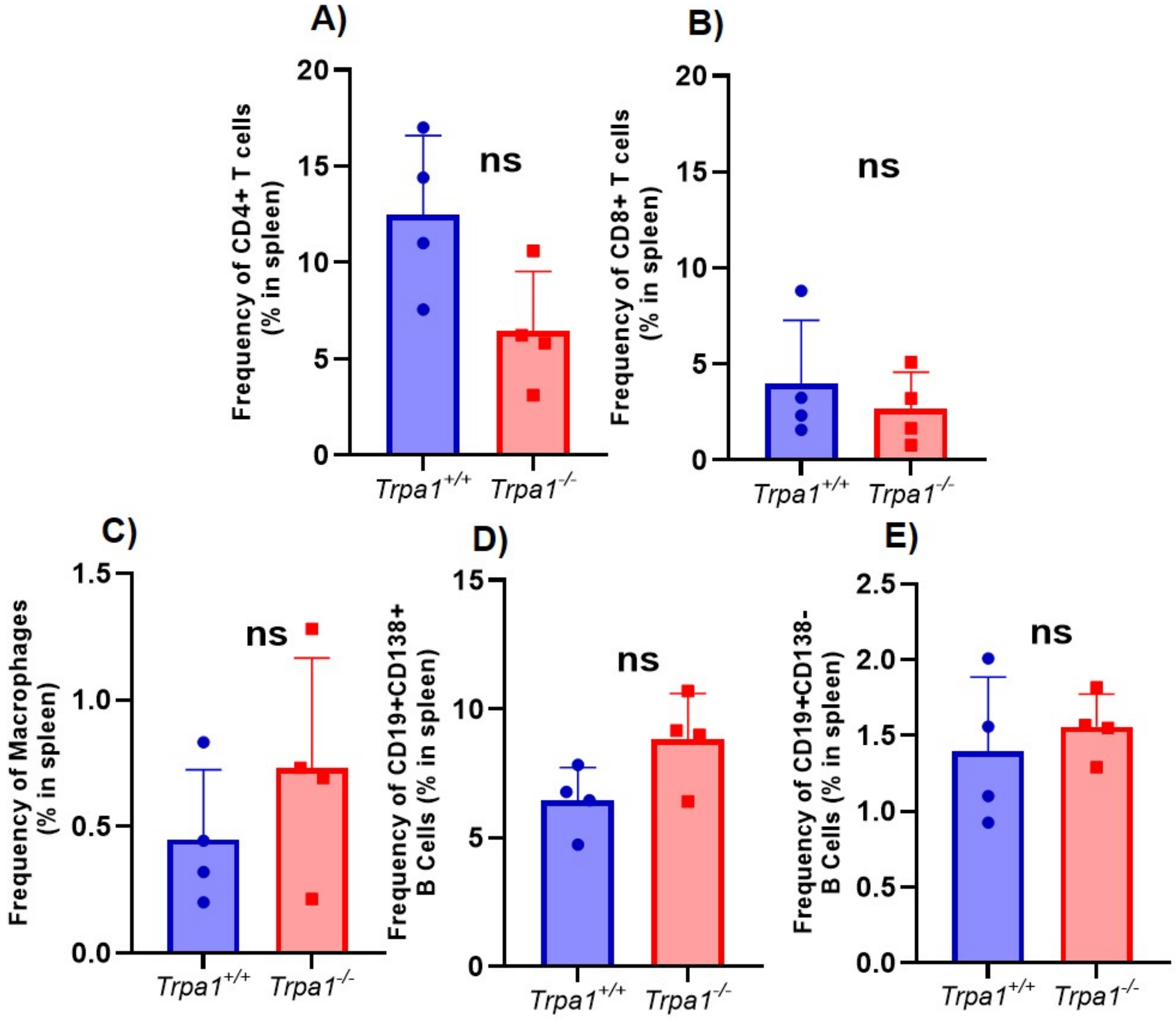

Supplementary Figure 3. Evaluation of immune cell populations in spleen of tumor-inoculated *Trpa1*$^{+/+}$ or *Trpa1*$^{-/-}$ mice. (A) CD4+ T lymphocytes; (B) CD8+ T lymphocytes; (C) Macrophages; (D) CD19+CD138+ B cells; (E) CD19+CD138- B cells. Statistical analyses were performed by Student's *t*-test. Values are presented as the mean (n = 4) ± SD of the frequency (%) in each group. ns=not significant.

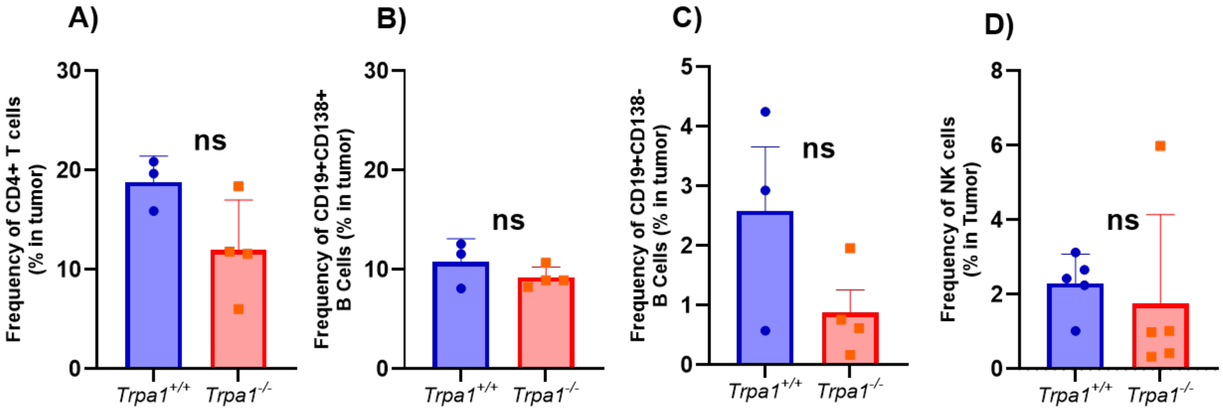

Supplementary Figure 4. Evaluation of immune cell populations in tumor stroma of *Trpa1*$^{+/+}$ or *Trpa1*$^{-/-}$ mice. (A) CD4+ T cells; (B) CD19+CD138+ B cells; (C) CD19+CD138- B cells; (D) Natural killer (NK) cells. Statistical analyses were performed by Student's *t*-test. Values are presented as the mean (n = 3 – 4) ± SD of the frequency (%) in each group. ns=not significant.

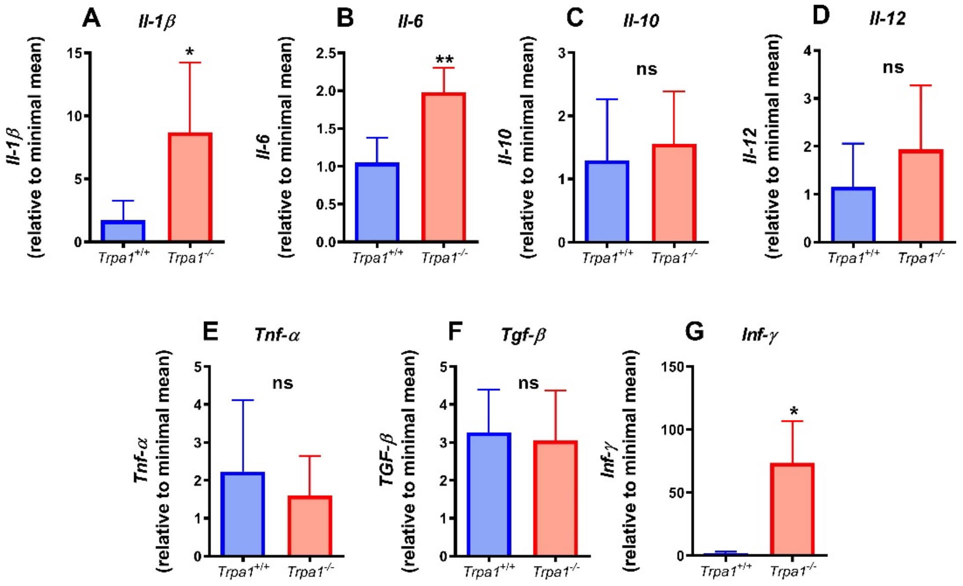

Supplementary Figure 5. Expression of inflammatory genes in the tumor stroma of *Trpa1*$^{+/+}$ and *Trpa1*$^{-/-}$ mice. (A) *Il-1β*; (B) *Il-6*; (C) *Il-10*; (D) *Il-12*; (E) *Tnf-α*; (F) *Tgf-β*; (G) *Ifn- γ*. Statistical analyses were performed by Student's *t*-test. * $p < 0.05$; ** $p < 0.01$. Values are presented as the mean (n = 4 – 5) ± SD (%) in each group. ns=not significant.
